# Inflammasomes in neurodegenerative diseases

**DOI:** 10.1186/s40035-024-00459-0

**Published:** 2024-12-23

**Authors:** Qianchen Wang, Songwei Yang, Xuan Zhang, Shanshan Zhang, Liping Chen, Wanxue Wang, Naihong Chen, Jiaqing Yan

**Affiliations:** 1 Department of Pharmacy, The First Affiliated Hospital of China Medical University, Shenyang, 110001 China; 2 Hunan Engineering Technology Center of Standardization and Function of Chinese Herbal Decoction Pieces, School of Pharmacy, Hunan University of Chinese Medicine, Changsha, 410208 China; 3 Department of Pharmacy, National Cancer Center/National Clinical Research Center for Cancer/Cancer Hospital, Chinese Academy of Medical Sciences and Peking Union Medical College, Beijing, 100021 China; 4 China Three Gorges University College of Medicine and Health Sciences, Yichang, 443002 China; 5 State Key Laboratory of Bioactive Substances and Functions of Natural Medicines, Institute of Materia Medica and Neuroscience Center, Chinese Academy of Medical Sciences and Peking Union Medical College, Beijing, 100050 China

**Keywords:** Inflammasome, Neurodegenerative diseases, Neurodegeneration, Neuroinflammation, Microglia

## Abstract

Inflammasomes represent a crucial component of the innate immune system, which respond to threats by recognizing different molecules. These are known as pathogen-associated molecular patterns (PAMPs) or host-derived damage-associated molecular patterns (DAMPs). In neurodegenerative diseases and neuroinflammation, the accumulation of misfolded proteins, such as beta-amyloid and alpha-synuclein, can lead to inflammasome activation, resulting in the release of interleukin (IL)-1β and IL-18. This activation also induces pyroptosis, the release of inflammatory mediators, and exacerbates neuroinflammation. Increasing evidence suggests that inflammasomes play a pivotal role in neurodegenerative diseases. Therefore, elucidating and investigating the activation and regulation of inflammasomes in these diseases is of paramount importance. This review is primarily focused on evidence indicating that inflammasomes are activated through the canonical pathway in these diseases. Inflammasomes as potential targets for treating neurodegenerative diseases are also discussed.

## Introduction

Neurodegenerative diseases are a common cause of death and morbidity worldwide, especially among the elderly population [1]. The prevalence of age-dependent diseases is on the rise. Neurodegenerative diseases including Alzheimer’s disease (AD), Parkinson’s disease (PD), Huntington’s disease (HD), and amyotrophic lateral sclerosis (ALS) cause impairments of memory, cognition, and motor function. Currently, there is no cure for these diseases, and existing treatments can only control symptoms or halt the progression of the disease [2]. Neurodegenerative diseases are characterized by the progressive accumulation of specific proteins in various regions of the brain, including alpha-synuclein (α-Syn) inclusions, tau neurofibrillary tangles, TAR DNA binding protein-43 (TDP43) inclusions, and amyloid plaques, accompanied by neuronal loss, vascular lesions, and gliosis. The various protein deposits coexist and are not specific. Furthermore, multiple protein deposits also occur simultaneously in subjects with neurodegenerative diseases and healthy individuals. The interaction of different proteins occurs through various mechanisms, which often accelerates the deposition process [3–5]. Although these diseases each have specific protein aggregates, it is increasingly clear that there is an interaction between beta-amyloid (Aβ), tau, and α-Syn, which may promote the occurrence and development of the disease through various molecular mechanisms. For example, the interaction between Aβ and tau may result in the activation of inflammation-related components, including the Nod-like receptor and pyrin domain-containing protein 3 (NLRP3) inflammasome and Toll-like receptors (TLRs), which in turn triggers neuroinflammation [6]. Although the precise pathogenesis of neurodegenerative diseases remains elusive, a growing body of evidence suggests that complex interactions between genetic, epigenetic, and environmental factors may play a role [7]. Nevertheless, to date, no effective treatment methods have been developed to slow, halt, or prevent the progression of any neurodegenerative diseases.

The occurrence and development of neurodegenerative diseases is accompanied by the involvement of the immune system and relevant immune cells in the brain. In the early stages of many neurological diseases, immune cells provide protection; however, their chronic activation can result in the spread of neuroinflammation, which ultimately leads to harmful changes within the brain [8]. Furthermore, persistent inflammation can result in the development of pathological changes in the affected tissue, indicating that the inflammatory stimuli are either not being terminated or that the normal resolution mechanisms are not functioning properly. Persistent stimuli may arise from environmental factors or the formation of endogenous factors (such as protein aggregates). They are perceived by the immune system as "foreign" or "danger" signals via pattern recognition receptors (PRRs) [9], which can recognize conserved pathogen-associated molecular patterns (PAMPs) or host-derived damage-associated molecular patterns (DAMPs). PRRs can be membrane-bound, enabling cells to monitor the extracellular space. They can also be intracellular, including nucleotide oligomerization domain (NOD)-like receptors (NLRs) and absent in melanoma 2 (AIM2)-like receptor (ALRs), which act as receptors for the inflammasome complex [10]. Inflammasomes are a key component of innate immunity [11]. Evidence from animal models, clinically relevant models, cell cultures, and human tissues suggests that activation of various inflammasomes is closely related to the pathogenesis of neurodegenerative diseases. In this review, we will discuss the roles of inflammasomes in neurodegenerative diseases, providing insights into the treatment of these diseases by targeting inflammasomes.

## Biological concept of inflammasomes

**Fig. 1:**
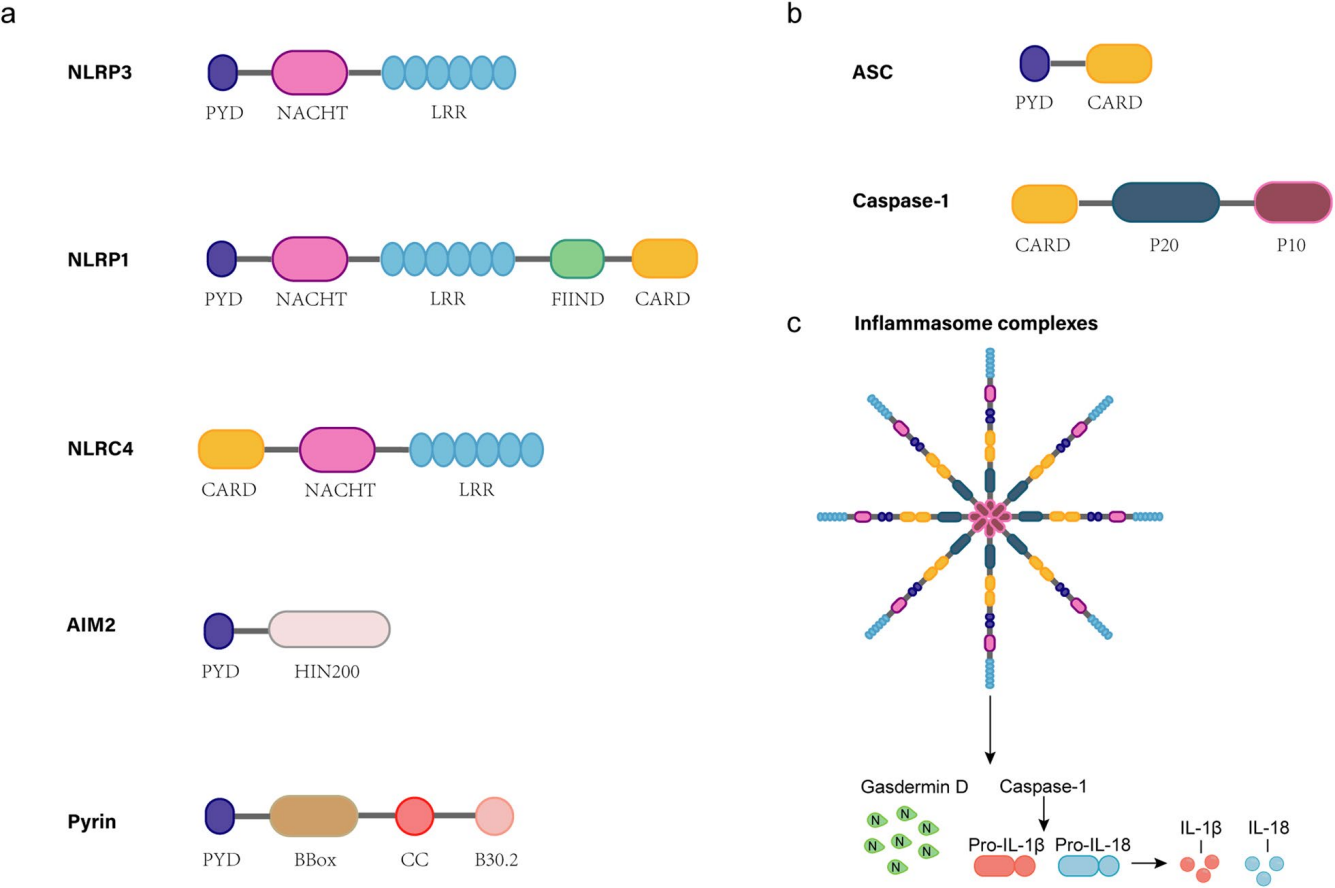
Structure of human inflammasomes and inflammasome complexes. **a** Schematic of compositions of NLRP3, NLRP1, NLRC4, AIM2 and Pyrin. **b** Apoptosis-Associated Speck-Like Protein (ASC) is composed of a PYD and a CARD. Caspase-1 consists of a CARD and a middle portion that includes the p20 and p10 subunits. **c** Upon inflammasome activation by various stimuli, homotypic PYD-PYD and CARD-CARD interactions facilitate the recruitment of ASC and the activation of caspase-1. Ultimately, oligomerization leads to the formation of the inflammasome complex, producing active caspase-1, which cleaves pro-IL-1β and pro-IL-18 into mature IL-1β and IL-18, and cleaves gasdermin D, forming pores to release cytokines, leading to pyroptosis. AIM2, absent in melanoma 2; CARD, caspase recruitment domain; FIIND, function-to-find domain; HIN-200, hemopoietic IFN-inducible nuclear proteins with 200-amino acid motif; LRR, leucine-rich repeat sequences; NACHT: NAIP (neuronal apoptosis inhibitor protein), CIITA (class II transactivator), HET-E (invariant chain of the MHC class II), and TP1 (telomerase-associated protein); NLRC4: Nod-like receptor and caspase recruitment domain-containing protein 4; NLRP: Nod-like receptor and pyrin domain-containing protein; PYD, pyrin domain

Inflammasomes are large, multi-protein complexes (Fig. 1) with a primary function of regulating the activation of the protease caspase-1. Caspase-1, in turn, regulates the proteolytic maturation of interleukin (IL)-1β and IL-18, as well as pyroptosis, a rapid, detrimental, and inflammatory form of cell death [12]. The assembly of inflammasomes is dependent upon the recognition of PAMPs or DAMPs via PRRs. PAMPs include unique microbial structures, such as microbial nucleic acids, bacterial secretion systems, and components of the microbial cell wall. DAMPs include monosodium urate crystals, ATP, or endogenous danger signals released by damaged or dying cells [13]. Based on the subcellular localization, PRRs can be divided into two major categories, membrane-bound and intracellular. TLRs and C-type lectin receptors are present on the plasma membrane and in endosomes. They are capable of recognizing extracellular PAMPs and DAMPs. The intracellular PRRs include retinoic acid-inducible gene-I (RIG-I)-like receptors, ALRs, and NLRs [13, 14]. A key node in PRR signal transduction is the activation of caspase family members, especially caspase-1. The central role of caspase-1 is to cleave the precursors of IL-1β and IL-18 into biologically active IL-1β and IL-18, and also gasdermin D (GSDMD), leading to inflammatory responses and pyroptosis [15]. The classic inflammasome complex is composed of a cytosolic sensor, which can be either NLR or ALR, an adaptor (apoptosis-associated speck-like protein containing CARD [ASC]), and an effector caspase-1 [16]. The NLR family is defined by the presence of a central nucleotide-binding and oligomerization (NACHT) domain, which is typically flanked by C-terminal leucine-rich repeats (LRRs) and N-terminal caspase activation and recruitment domain (CARD) or pyrin domain (PYD). The LRRs are believed to be involved in ligand sensing and autoregulation, while the CARD and PYD mediate homotypic protein–protein interactions for downstream signaling. The NACHT domain is the sole domain shared by all NLR family members and is capable of activating the signaling complex through ATP-dependent oligomerization [16]. Assembly of the inflammasome results in the recruitment and activation of caspase-1. The activation of caspase-1 is initiated by its self-cleavage and conformational change, a process that is typically facilitated by the adaptor protein ASC [17]. ASC is a bipartite molecule comprising an N-terminal PYD and a C-terminal CARD. This allows it to bridge the sensor (NLRs or ALRs) and the effector pro-caspase-1. Pro-caspase-1 is then activated, resulting in the cleavage of pro-IL-1β and pro-IL-18 into mature, biologically active cytokines. Studies have demonstrated that NLRP3 and AIM2 contain a PYD that does not directly interact with caspase-1, but interacts with the PYD of ASC. The CARD domain of ASC binds to the CARD of caspase-1 through CARD-CARD interactions. Consequently, ASC represents a fundamental component of numerous inflammasomes [12, 18].

**Fig. 2:**
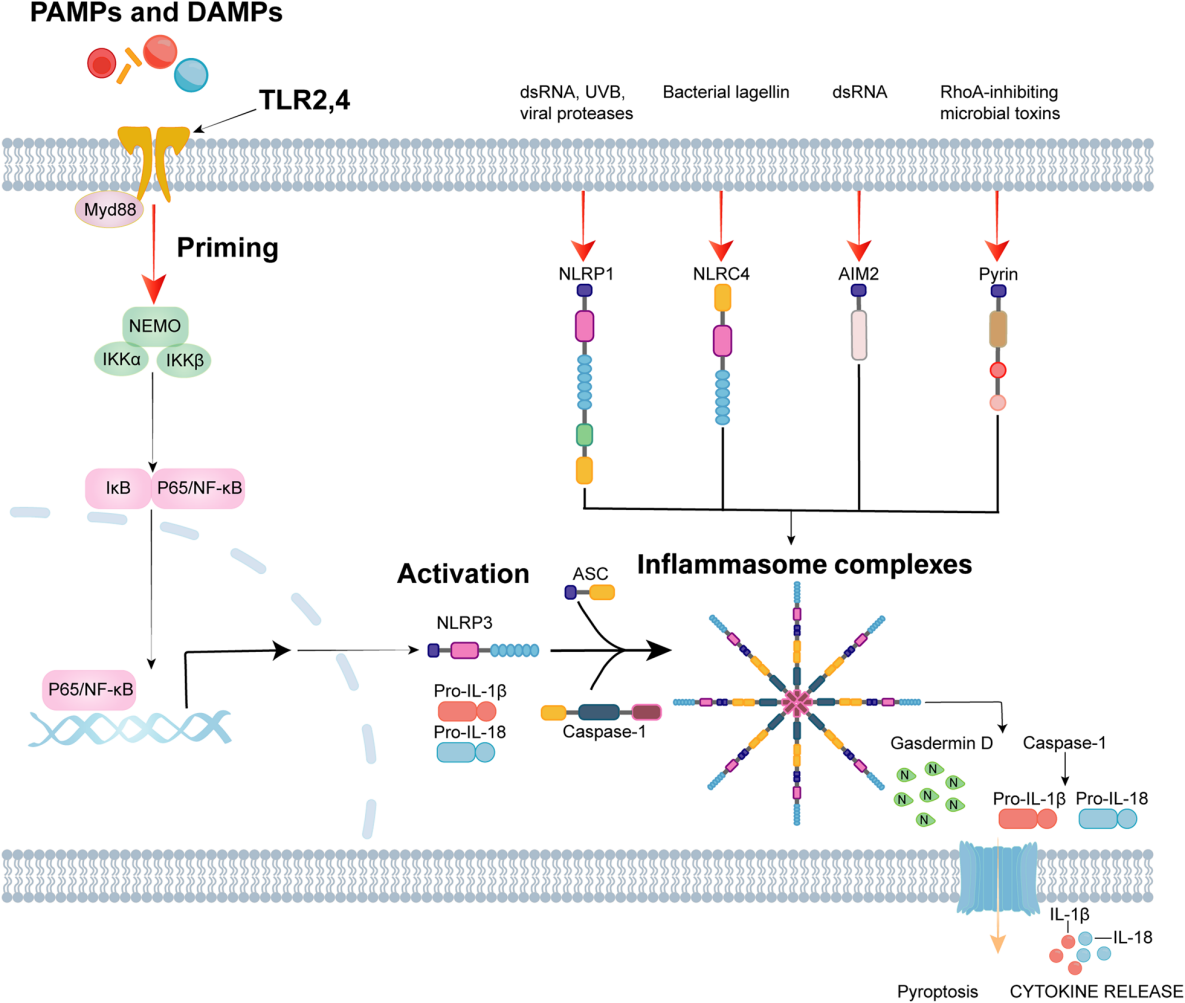
Inflammasome activation in neurodegenerative diseases. In neurodegenerative diseases, pathogen-associated molecular patterns (PAMPs) or host-derived damage-associated molecular patterns (DAMPs) activate Toll-like receptors (TLRs). TLR activation induces expression of NLRP3, pro-IL-1β, and pro-IL-18 through nuclear factor-kB (NF-kB) transcription. The intracellular NLRP3 is activated by pathological proteins or DAMPs, NLRP1 recognizes dsRNA and viral proteases, NLRC4 identifies bacterial flagellin, AIM2 is specifically activated by dsDNA, and Pyrin recognizes RhoA-inhibiting microbial toxins. Following priming, the inflammasome undergoes activation upon perception of endogenous or exogenous signals, which in turn promote the recruitment of ASC and activation of caspase-1, leading to the formation of an inflammasome complex. Subsequently, the active caspase-1 cleaves pro-IL-1β and pro-IL-18 into mature IL-1β and IL-18, and cleaves gasdermin D, forming membrane pores to release cytokines, thereby initiating pyroptosis. AIM2, absent in melanoma 2; NLRC4: Nod-like receptor and caspase recruitment domain-containing protein 4; NLRP1: Nod-like receptor and pyrin domain-containing protein 1; NLRP3: Nod-like receptor and pyrin domain-containing protein 3

The initiation of inflammasome activation varies among different cell types, sensors, stimuli, and pattern recognition (Fig. 2). It has been demonstrated that transcription factors, such as nuclear factor-kappa B (NF-κb), can regulate the expression of inflammasome components, including NLRP3, thereby influencing the assembly and activity of the inflammasome [19]. Post-translational modifications of inflammasomes can also affect their activity, including phosphorylation, ubiquitination, acetylation, alkylation, S-nitrosylation, and S-glutathionylation, among others. These modifications can affect the stability, conformation, interactions, and subcellular localization of inflammasome components, thereby regulating the activation and inhibition of the inflammasome in a finely tuned manner [20]. Interactions between proteins can significantly influence the assembly and activation of inflammasomes. For instance, adaptor proteins such as ASC, can facilitate the aggregation of inflammasome components, while inhibitory proteins can impede this process [21]. Some signaling pathways, such as the TLR signaling pathway, often cross-talk with the inflammasome pathway. This cross-regulation can either enhance or inhibit inflammasome activation [22, 23]. It has been demonstrated that metabolic byproducts within cells, such as fatty acids and intermediates of energy metabolism, can also regulate inflammasome activation. Alterations in intracellular metabolic byproducts can impact the modification state of inflammasome components, thereby affecting the inflammatory response [24]. Genetic polymorphisms, mutations, and epigenetic modifications also play a role in regulating the activity of inflammasomes. For example, mutations of the *MEFV* gene may lead to inflammasome activation, while epigenetic modifications such as DNA methylation and histone modifications can influence the expression of genes associated with inflammasomes [25, 26]. The regulation of inflammasomes is a complex process that involves a multitude of molecular mechanisms and biological pathways.

## Neuroinflammation

**Fig. 3:**
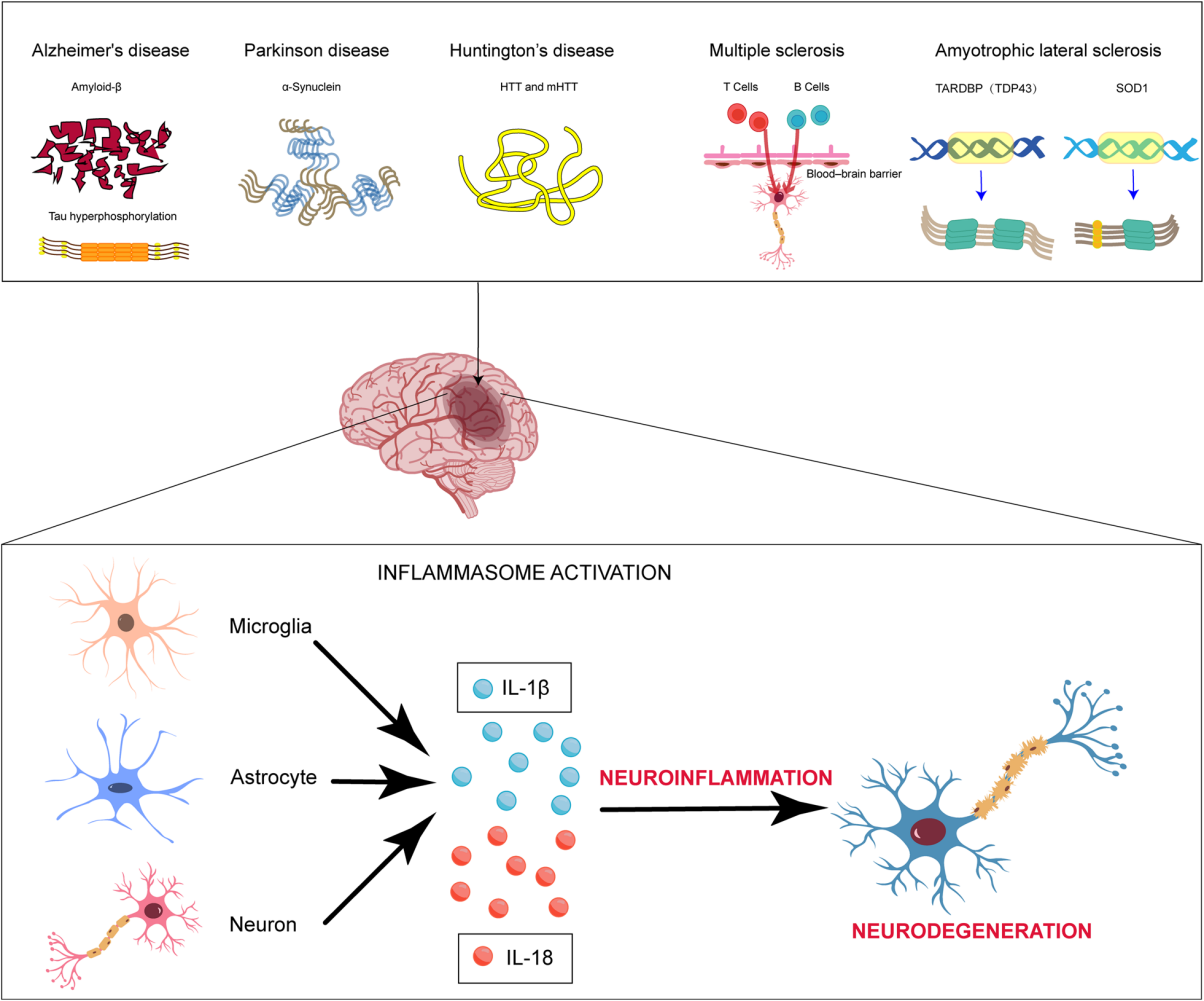
Neuroinflammation caused by inflammasome activation. Inflammasome activation has been observed in numerous neurodegenerative diseases, the majority of which are attributed to aberrant aggregation of misfolded proteins, resulting in inflammasome activation. In Alzheimer’s disease, the pathological hallmarks are the deposition of beta-amyloid protein and hyperphosphorylation of tau protein. In Parkinson’s disease, the underlying mechanism involves the aberrant aggregation of alpha-synuclein (α-Syn). In Huntington’s disease, the underlying cause is the aberrant aggregation of the huntingtin protein (HTT) and the mutated form of this protein, mHTT. In amyotrophic lateral sclerosis, the underlying mechanism involves the aberrant aggregation of TDP-43 and SOD1 proteins. In multiple sclerosis, peripheral immune cells, namely T cells and B cells, infiltrate the central nervous system through the damaged blood–brain barrier, thereby activating inflammasomes. Following inflammasome activation, active caspase-1 cleaves pro-IL-1β and pro-IL-18, resulting in the production of mature IL-1β and IL-18. These mature cytokines serve as principal mediators of chronic inflammation in neurodegenerative diseases. Moreover, active caspase-1 also cleaves gasdermin D, resulting in the formation of pores on the cell membrane, leading to release of cellular contents into the extracellular environment and induction of pyroptosis

Neuroinflammation is defined as the inflammatory response that occurs in the central nervous system (CNS). This response can be triggered by a variety of factors, including infection, autoimmune diseases, oxidative stress, neurodegenerative diseases, etc. [27]. Neuroinflammation is characterized by the production of pro-inflammatory cytokines, including IL-1β, IL-6, IL-18, pleiotropic cytokine IL-10, tumor necrosis factor (TNF), and interferon-gamma (IFN-γ), by innate immune cells of the CNS. Small-molecule messengers, including prostaglandins, nitric oxide (NO), chemokines including macrophage inflammatory protein-1 alpha and granulocyte colony-stimulating factor, and reactive oxygen species (ROS) [28–30], are also involved in neuroinflammation. IL-1β and IL-18 released via inflammasome activation lead to chronic inflammation (Fig. 3). Studies have demonstrated that neuroinflammatory diseases, particularly those that have progressed to the neurodegenerative stage, exhibit characteristics of cellular senescence. Cellular senescence is defined as a stable cell cycle arrest, in which cells are unable to proliferate despite optimal growth conditions and stimuli. Cellular senescence is associated with a variety of human diseases. Preliminary data indicate that cellular senescence is present in neuroinflammatory diseases, suggesting that it may be a potential driver of pathogenesis [28].

Microglia are the primary immune cells in the CNS and play a crucial role in neuroinflammation. In pathological conditions, microglia are activated and undergo a phenotypic transition to a pro-inflammatory state, releasing inflammatory factors that lead to local or systemic neuroinflammation. Astrocytes also undergo reactive proliferation in response to neuroinflammation, known as astrogliosis. In this process, astrocytes release inflammatory mediators and participate in the repair of cellular damage [31]. However, astrocytes can transform into the neurotoxic A1 type. The A1 astrocytes may promote the entry of immune cells into the CNS by secreting neurotoxins, complement components, and chemokines, thereby exacerbating neuroinflammation and neuronal damage [32]. Moreover, classically activated neuroinflammatory microglia can induce the generation of A1-type reactive astrocytes [33]. Microglial activation represents a significant contributing factor in the pathogenesis of neuroinflammation. Microglia rapidly respond to imbalances in the internal environment of brain by releasing a variety of pro-inflammatory and cytotoxic components, leading to neuroinflammation and neurodegeneration [28]. It is noteworthy that the association between microglia and astrocytes is relatively weak. However, studies have demonstrated that the phenotypic transition of microglia requires signals from astrocytes, rather than from microglia themselves. This further emphasizes the significance of the astrocyte-microglia crosstalk in neuroinflammation [34]. In addition, directly blocking the microglia-mediated transformation of astrocytes into the A1 neurotoxic phenotype leads to neuroprotective effects. This transformation may occur in a variety of neurodegenerative diseases [35]. Research has demonstrated that disease-associated microglia (DAM) play a pivotal role in the pathogenesis of neurodegenerative diseases and brain aging. DAM is a recently identified subset of resident macrophages found at sites of neurodegeneration. DAM express multiple genes related to AD and other neurodegenerative diseases, including triggering receptor expressed on myeloid cells 2 (TREM2), a receptor essential for DAM activation [36, 37]. Aggregation of misfolded proteins is a defining feature of numerous neurodegenerative diseases and can result in cytotoxicity. In AD, abnormal aggregation of Aβ can act as DAMPs, activating inflammasomes within microglia, resulting in the release of inflammatory factors and further exacerbating neuroinflammation and neurodegenerative processes [37].

Furthermore, autophagy is crucial for maintaining the stability of the intracellular environment and cellular health, as it is involved in the clearance of damaged proteins and organelles. Impaired autophagy can result in the accumulation of misfolded proteins and aggregates within neurons, leading to neuronal dysfunction and death [38]. Moreover, during the development of neurodegenerative diseases, inflammasome activation can promote the initiation of autophagy, which helps clear intracellular pathogens and damaged organelles, thereby reducing inflammatory responses. The activation of autophagy can in turn regulate the activity of inflammasomes, affecting the release of inflammatory factors. The dual regulation balances the host defense inflammatory response and prevents excessive inflammation [39, 40]. Therefore, autophagy exerts a regulatory effect on inflammasome activation.

## Inflammasomes in neurodegenerative diseases

### Inflammasomes in AD

AD is the most prevalent form of chronic neurodegenerative disease and the most common type of dementia, primarily affecting the elderly population. The pathological characteristics of AD include brain atrophy, Aβ plaques, neurofibrillary tangles (aggregates of tau protein), neuroinflammation, and loss of neurons and synapses [41]. In addition to the aforementioned pathological characteristics, AD patients exhibit a reduced Aβ42 level in cerebrospinal fluid (CSF) and increased levels of markers of oxidative stress. Concurrently, inflammatory cytokines IL-1β and TNF-α are elevated, and the levels of NLRP1, NLRP3, ASC, cleaved caspase-1, IL-1β, and GSDMD are also increased, co-localized with Aβ plaques [42, 43]. This evidence suggests that, in addition to the amyloid hypothesis, neuroinflammation is also a key component of the pathogenesis of AD.

Inflammasomes have been demonstrated to play a role in AD pathogenesis. Peripheral monocytes of AD patients show increased mRNA expression of NLRP1, NLRP3, PYCARD, caspase-1, IL-1β, and IL-18, and activation of the NLRP1 and NLRP3 inflammasomes [44]. In early stages of AD, levels of inflammasome proteins in neurons and microglia are increased. NLRP1 is mainly expressed in neurons, while NLRP3 is mainly found in microglia. Caspase-1 is present in the parenchyma of the hippocampus, while ASC is expressed in both neurons and microglia [43]. Furthermore, some studies have demonstrated that Nlrp3 or Caspase-1 knockout in mice carrying familial AD-related mutations results in a notable protection from spatial memory loss and other sequelae associated with AD, evidenced by reduced activation of caspase-1 and IL-1β in the brain and enhanced Aβ clearance. In the APP/PS1 AD mouse model, NLRP3 inflammasome activation results in the transformation of microglia from the M2 type (which promotes repair and clearance) to the M1 type (which is pro-inflammatory and neurotoxic), thereby reducing Aβ clearance and promoting its deposition [45]. Aβ and tau aggregates as DAMPs can activate toll-like receptor 4 (TLR4), which subsequently initiates signal transduction through MyD88-dependent and TRIF-dependent pathways. The pro-inflammatory cytokines produced (such as IL-1β) can further promote assembly and activation of the NLRP3 inflammasome through the IL-1R/MyD88 pathway [46, 47].

NLRP3 inflammasome activation in AD is a complex process involving multiple intracellular signaling pathways and external stimuli. Aβ activates Syk and inactivates downstream adenosine 5’-monophosphate-activated protein kinase (AMPK), which causes excessive mitochondrial fission, leading to NLRP3 inflammasome activation in microglia. This results in cognitive decline, amyloid plaques, and neurofibrillary tangles. In this process, the microglial receptor TREM2 may be involved in the activation of Syk by Aβ and the induction of NLRP3 inflammasome activation, as TREM2 has an ITAM motif that activates Syk and Aβ has been reported to bind directly to TREM2. TREM2 can also maintain the energy and anabolic metabolism of microglia through the mammalian target of rapamycin (mTOR) signaling pathway, thereby reducing inflammation [48, 49]. Meanwhile, insoluble fibrils of endogenous Aβ abnormally aggregate in the brains of AD patients, resulting in the loss of synapses and neurons. These fibrils also interact with multiple receptors on microglia to activate inflammasomes. Notably, the fibrillar state of Aβ is a necessary condition for microglial release of IL-1β [50]. Once Aβ fibrils are phagocytosed by microglia, they are transported to lysosomes, where they cause damage to the lysosomal membrane, resulting in the release of lysosomal protease cathepsin B into the cytoplasm, which then activates the inflammasomes [51]. The association between cathepsin B and AD pathogenesis has been confirmed. It is hypothesized that cathepsin B regulates the migration of microglia through PI3K-Akt signaling, acting as an intermediary among phagocytosis of Aβ, lysosomal damage, and release of IL-1β by microglia. Other phagosomal factors, including other cathepsins, may also participate in inflammasome activation [48]. CD36, a PRR, facilitates the intracellular transition of soluble endogenous Aβ to particulate ligands, which results in lysosomal rupture and subsequent NLRP3 inflammasome activation [52]. CD36 may act as a receptor for soluble Aβ, transmitting signals from Aβ to inflammasomes, possibly through the NF-κB pathway, which may result in increased expression of IL-1β and IL-18 [53]. The P2X7 receptor is an ion channel receptor that can be activated by extracellular adenosine triphosphate (ATP), resulting in the opening of ion channels. This causes the efflux of potassium ions (K^+^) and the influx of sodium ions (Na^+^) and calcium ions (Ca^2^^+^), thereby activating the NLRP3 inflammasome and exacerbating neuroinflammation. Studies have demonstrated that activation of the P2X7 receptor may facilitate the deposition of Aβ and aberrant phosphorylation of tau protein, thereby accelerating the pathological progression of AD [54, 55].

One of the principal neuropathological characteristics of AD is the formation of NFTs, caused by aberrant aggregation of tau protein. Tau is a microtubule-associated protein that stabilizes the microtubule structure within nerve cells. In AD, tau protein undergoes aberrant phosphorylation, resulting in loss of function and aggregation to form NFTs. This process leads to inflammasome activation and subsequent induction of neuroinflammation [56]. In transgenic mice with tauopathy and in hippocampal samples from AD patients, the level of NLRP3 acetylation is elevated, which is associated with inflammasome activation. In cellular models and transgenic mice, researchers have confirmed that tau protein can directly acetylate NLRP3, particularly at the lysine residues K21, K22, and K24 in its PYD domain, thereby inducing activation of the inflammasomes. Furthermore, overexpression of tau protein in the hippocampal CA1 neurons of mice results in impaired cognitive function, transmission of tau to microglia, and activation of microglia accompanied by NLRP3 acetylation and inflammasome activation [57]. Tau seeds are internalized by microglia and transported to lysosomes. Once inside the lysosomes, tau seeds may cause lysosomal rupture, releasing contents including cathepsin B. The released cathepsin B and other DAMPs activate the NLRP3 inflammasome, resulting in aggregation of ASC and subsequent activation of caspase-1. Furthermore, the activated caspase-1 cleaves pro-IL-1β into active IL-1β. This process ultimately exacerbates tau pathology, including both endogenous and non-endogenous seed-induced tau pathology [58]. In the Tau22 mouse model, researchers have found evidence of NLRP3 inflammasome activation, namely, increased expression levels of cleaved caspase-1, IL-1β, and ASC. This suggests that the aberrant tau protein facilitates assembly and activation of the NLRP3 inflammasome. In Tau22 mice, injection of Aβ-containing brain homogenate into the hippocampus induced tau pathology, whereas in the inflammasome-knockout Tau22 mice, this was not observed, suggesting that the NLRP3 inflammasome is involved in Aβ-induced tau pathology in AD [59, 60]. It is noteworthy that Aβ plaques and tau protein are not only individually implicated in the pathological process of AD, but may also serve a facilitating role. A previous study demonstrated that Aβ plaques facilitate the rapid expansion of AD-brain-derived pathological tau protein into large tau aggregates and promote the formation of tau aggregates (NP tau) around degenerating neurites surrounding Aβ plaques. This evidence indicates that Aβ facilitates tau protein pathology in AD by promoting tau protein aggregation [61].

A growing body of evidence indicates that the gut-brain axis plays a pivotal role in the pathogenesis of AD. Research has demonstrated that the gut microbiota of AD patients can produce amyloid peptides, which accumulate in the brain through the gut-brain axis. Furthermore, administration of the gut microbiota from AD patients exacerbates AD pathology and cognitive dysfunction in 3 × Tg mice. Imbalances of metabolites in the gut, such as short-chain fatty acids and inflammatory polyunsaturated fatty acid metabolite, stimulate the maturation of microglia, thereby activating inflammasomes. Various strategies to regulate the composition of the gut microbiota have been demonstrated to alleviate cognitive impairments. This evidence suggests that a dysbiotic gut microbiota can lead to inflammasome activation and contribute to the pathogenesis of AD [62–64].

NLRP3 inflammasome activation exacerbates the progression of AD; consistently, several genes related to AD are highly expressed in microglia, including *INPP5D* (inositol Polyphosphate-5-phosphatase D) and *CALHM2* (calcium Homeostasis Modulator 2). *INPP5D* encodes the Src homology 2-containing inositol phosphatase-1 (SHIP1) protein. SHIP1 regulates the PI3K/Akt signaling pathway by dephosphorylating phosphatidylinositol phosphates (PIPs), thereby regulating the phagocytic function and immune response of microglia. Studies have demonstrated decreased protein level and increased mRNA expression of INPP5D in the brains of AD patients. Further validation demonstrated that reducing INPP5D activity induces formation of the NLRP3 inflammasome, cleavage of caspase-1, and secretion of IL-1β and IL-18 [65, 66]. Calhm2 is a member of the calcium homeostasis regulator family. Research has demonstrated increased expression level of Calhm2 in AD mouse models. Moreover, *Calhm2*-knockout mice exhibit significantly reduced Aβ deposition, neuroinflammation, and cognitive impairment. This indicates that inhibition of microglial activation may attenuate inflammasome activation and thereby alleviate the pathology of AD [67]. In conclusion, NLRP3 inflammasome activation may contribute to the progression of AD via production of pro-inflammatory cytokines such as IL-1β and reduction of Aβ and tau clearance, leading to increased deposition and formation of a self-perpetuating cycle [53].

In addition to the NLRP3 inflammasome, other inflammasomes have also been implicated in AD. Recent studies have demonstrated elevated mRNA levels of *NLRP1* in individuals diagnosed with severe AD. NLRP1 is localized to CD14 cells, and the proportion of CD14^+^/NLRP1^+^ immune cells is also higher in these patients, indicating that NLRP1 is activated in AD [43, 68]. Another study demonstrated that the NLRP1 inflammasomes are expressed in primary neurons of the CNS. In response to stress, the NLRP1 inflammasomes are activated, leading to activation of caspase-1 and subsequent caspase-6 activation, which is associated with AD. This results in axonal degeneration [69]. Similarly, aged mice, particularly 24-month-old mice, show significant increases of neuronal injury, inflammatory cytokines, and NLRP1 inflammasomes [70]. Activation of the NLRP1 inflammasomes in APP/PS1 mice results in increased Aβ production, neuroinflammation, and dysfunction of the AMPK/mTOR signaling pathway related to autophagy at 9 months of age [71]. Genetic knockout of NLRP1, Caspase-1, or Caspase-6 restores the episodic memory and spatial learning of J20 mice, increases the density of dendritic spines in the hippocampal CA1 region, and normalizes the levels of synaptophysin in the hippocampal dentate gyrus and CA3 region [72].

A previous study employed streptozotocin to induce memory deficits and cognitive decline in mice and observed an increase in Nodlike receptor and caspase recruitment domain-containing protein 4 (NLRC4) in hippocampal samples [73]. However, another study observed that in the hippocampus of aged APP/PS1 mice, the expression of ASC protein was increased, yet there were no notable alterations in NLRP1, NLRP3, or NLRC4 [74]. In mice with Aβ_1-42_-induced AD, the expression level of AIM2 increases, predominantly in microglia. Specific knockout of AIM2 in microglia improves cognitive function and synaptic plasticity in mice [75, 76]. Similarly, activation of the AIM2 inflammasomes may exacerbate neurodegeneration by promoting Aβ deposition and abnormal phosphorylation of tau protein, leading to an inflammatory response that further affects cognitive function [77]. A deeper understanding of the specific contributions of various inflammasomes to AD pathogenesis could facilitate the development of targeted therapies that modulate inflammasome activity to reduce inflammation and potentially slow the progression of AD. Further research is needed to elucidate the mechanisms by which these inflammasomes are activated and to determine how their activation affects the course of the disease.

### Inflammasomes in PD

PD is the second most prevalent neurodegenerative disorder only next to AD, characterized by the loss of dopaminergic neurons in the substantia nigra pars compacta (SNpc) and the presence of Lewy bodies mainly composed of abnormally aggregated α-Syn [78, 79]. In the early stages of PD, prior to the formation of Lewy bodies, the loss of neurons in the SNpc has already occurred, along with oxidative stress and autophagy impairment [79]. The abnormal aggregation of α-Syn activates the NLRP3 inflammasome in microglia through interaction with TLRs, leading to release of pro-inflammatory cytokines through translocation of NF-κB, thereby damaging dopaminergic neurons [80]. Concurrently, aberrant aggregation of α-Syn may influence mitochondrial functionality, resulting in diminished ATP production and augmented ROS levels. This, in turn, affects lysosomal function, leading to the accumulation of protein aggregates [81]. The dysfunction of mitochondria and production of ROS may serve as the basis for NLRP3 inflammasome activation in microglia [82].

Studies have demonstrated that in the cerebral cortex of individuals diagnosed with PD and those without a PD diagnosis but with substantia nigra cell loss, there is an increase in the expression of inflammasome-associated proteins, including IL-1β, TNF-α, and NLRP3, in comparison to the control group [83]. Research has indicated that in the peripheral blood mononuclear cells (PBMCs) of patients with PD, there is increased gene expression of NLRP3, ASC, and caspase-1, as well as elevated protein levels of NLRP3, caspase-1, and IL-1β, indicating NLRP3 inflammasome activation in the PBMCs of PD patients[84]. Similarly, another study found elevated levels of α-Syn as well as inflammatory factors NLRP3, caspase-1, and IL-1β in the CSF of PD patients [85].

In vitro experiments exposing human brain-derived microglia and C57BL/6 J mouse brain-derived microglia to α-Syn monomers and fibrils revealed activation of the typical NLRP3 inflammasome and secretion of IL-1β [86]. NLRP3 inflammasome activation in PD may occur through direct recognition of α-syn aggregates by TLR2 and TLR4, with signal transduction leading to the activation of NF-κB. NF-κB then migrates to the nucleus, where it promotes the expression of various inflammatory factors, including NLRP3 and pro-IL-1β. Following NLRP3 inflammasome activation, caspase-1 is subsequently activated, resulting in the release of IL-1β and IL-18 [87]. TLR4-deficient mice have demonstrated an increased number of surviving dopaminergic neurons, a reduced number of activated glial cells, decreased accumulation of α-syn, as well as suppression of NF-κB and NLRP3 inflammasome activation [88]. Evidence indicates that in the 1-methyl-4-phenyl-1,2,3,6-tetrahydropyridine (MPTP)-induced mouse model of PD, NLRP3 inflammasome activation in microglia directly leads to neuronal death induced by MPTP. Furthermore, MPTP or its metabolite MPP^+^ may activate the NLRP3 inflammasome by producing mitochondrial ROS [89]. Similarly, following treatment with 6-hydroxydopamine, the levels of NLRP3 and ASC exhibited a significant increase [90]. These findings collectively indicate NLRP3 inflammasome activation in both in vitro models of PD induced by toxic substances and in PD patients. Studies have demonstrated that the use of NLRP3^–/–^ mice in the PFF model resulted in a significant reduction in the levels of ASC, caspase-1, and α-Syn [91]. Similarly, treatment of α-Syn mice with an NLRP3 inhibitor reduced the insoluble aggregation of α-Syn and ameliorated the loss of dopaminergic neurons [92].

The *SNCA* A53T mutation is associated with early-onset familial PD. This mutation increases the tendency of α-Syn to form toxic oligomers. The aggregation of A53T α-Syn and mitochondrial dysfunction together promote the activation of inflammatory bodies in PD [93]. Inhibiting the p38-TFEB pathway effectively reduces the NLRP3 inflammasome activation, thereby alleviating the motor and autophagy impairments observed in the A53T transgenic mouse model [94]. Research has demonstrated that in the adeno-associated virus overexpressing αSyn (AAV-α-Syn) model, stereotactic injection of AAV-α-Syn into the SNpc of Fyn^−/−^ mice resulted in significant reductions of microglial activation, formation of ASC specks, NLRP3 inflammasome activation and IL-1β secretion, compared to wild-type mice [95]. Moreover, serum/glucocorticoid receptor kinase 1 (SGK1) is found to be upregulated in the brains of PD patients. Inhibition of SGK1 in glial cells can correct the inflammatory characteristics of glial cells by inhibiting the NF-κB-, NLRP3 inflammasome-, and cGAS-STING-mediated inflammatory pathways [96]. Similarly, activation of nuclear factor erythroid 2-related factor 2 (Nrf2) and JWA (a tumor suppressor gene), and overexpression of BAG3 (Bcl2-associated athanogene 3) can inhibit the activation of NLRP3 and caspase-1, reduce the nuclear transfer of NF-κB, and thereby suppress inflammation [97–100]. These findings collectively suggest that modulation of inflammasome activation in PD can be achieved by activating or inhibiting upstream molecules.

The *PRKN* gene encodes the Parkin protein, which has been demonstrated to suppress neuroinflammation by ubiquitinating the NLRP3 inflammasome. When mitochondrial function is impaired, Parkin can facilitate their selective autophagy, which involves mitochondrial tagging and ultimately degradation by lysosomes [101]. In the absence or with inactivation of Parkin, NLRP3 accumulates in DA neurons, initiating inflammasome activation. Furthermore, the loss of Parkin activity results in increased ROS production by mitochondria through accumulation of another Parkin ubiquitination substrate, ZNF746/PARIS, thus promoting NLRP3 complex assembly [102].

In addition to NLRP3 inflammasome activation in PD, activation of other inflammasomes in PD remains a matter of debate. In the MPTP mouse model, the levels of NLRP1, AIM2, NLRP2, NLRP3, and NLRC4 are all elevated compared to the saline mouse group, with the NLRP3 increase being the most significant [103]. Additionally, AIM2 activation has been found to exert a protective effect against MPTP-induced behavioral deficits and neuroinflammation independent of the inflammasome in the MPTP mouse model [104]. However, in the PBMCs of PD patients, mRNA expression of NLRP3 is increased, while the mRNA expression of AIM2, NLRP1, and NLRC4 shows no significant changes [84]. Consequently, further research is required to ascertain whether other inflammasomes are activated in PD and the underlying mechanisms.

Some studies have demonstrated that microglial exosomes can facilitate the transfer of α-Syn between neurons, resulting in the aggregation and neurotoxicity of α-Syn [105]. In the CSF from PD patients, exosomes derived from microglia and macrophages contain monomeric α-Syn, which can induce α-Syn aggregation in neurons. A study injecting PD patient plasma-derived exosomes into the mouse brain found that these exosomes can spread throughout the brain [106]. It is of note that peripheral monocytes can phagocytose aggregated α-syn and lead to a more pronounced NLRP3 inflammasome response [107]. These α-Syn are likely to be carried by exosomes and transmitted to the periphery. Therefore, exosomes may spread inflammatory responses and activate NLRP3 inflammatory responses.

### Inflammasomes in HD

HD is an inherited neurodegenerative disorder caused by an abnormal expansion of the CAG repeat sequence in the huntingtin (*HTT*) gene. This expansion results in the production of a huntingtin protein with an excessively long polyglutamine (polyQ) tract [108]. In HD, both wild-type HTT and mutant HTT (mHTT) form aggregates and accumulate abnormally in the nucleus and processes of neurons, leading to the disruption of various cellular functions. Therefore, HTT and mHTT, particularly soluble mHTT in the CSF, can be used as biomarkers, which are positively correlated with the severity of symptoms [109]. Studies have shown that inflammatory cytokines such as IL-1β, IL-6, TNF-α, and MCP-1 are increased in the brains and CSF of HD patients. In addition, HD patients show signs of microglial and astrocyte activation in the brain [110]. Meanwhile, IL-6, IL-8, TNF-α, eotaxin-3, MIP-1β, and eotaxin levels are increased in the blood of HD patients [111]. In HD, the increased mRNA expression of IL-6, IL-1β, and TNF-α leads to microglial activation, which is intended to remove mHTT but instead exacerbates neurodegeneration [112, 113]. The presence of mHTT may also lead to disruption of the blood–brain barrier, potentially allowing peripheral immune cells to enter the CNS and exacerbate neuroinflammation [114]. Another study found that inhibition of TNF-α in the R6/2 HD transgenic mouse model could modulate neuroinflammation, caspase activation, mHTT aggregate burden, and motor dysfunction [115]. Using microarray and DeepSAGE technologies, *AIM2* in peripheral blood has been revealed to be a biomarker of HD, and AIM2 may be activated in HD [116]. However, current evidence is not sufficient to prove that the peripheral blood marker has the same significance as biomarkers within the brain, and the peripheral effects are not as profound as those within the neurons. Studies have found that the expression levels of NLRP3 and caspase-1 are increased in the striatal region in the R6/2 mouse brain [117]. Another study showed a significant increase in IL-1β and a decrease in IL-18 level in HD [118, 119]. This suggests that there are other mechanisms that regulate IL-18 level. The use of NLRP3 inhibitors in R6/2 mice reduces NLRP3 activation and ROS production, rescues neurons, and reduces microglial activation [120], which support the role of NLRP3 in HD. Activation of Nrf2 can increase the resistance of neurons to cytotoxicity caused by mHTT aggregates [121]. Meanwhile, administration of anti-inflammatory drugs celecoxib and meloxicam can attenuate the behavioral and biochemical changes in a rat model of quinolinic acid-induced HD [120]. However, in another study, acetylsalicylic acid and rofecoxib failed to show neuroprotective effects in the N171-82Q and R6/2 transgenic HD mouse models [122]. These results not only suggest that the activation of anti-inflammatory factors or the use of anti-inflammatory drugs may have therapeutic effects in HD, but also elucidate the roles of inflammasomes in HD. More studies are needed to find out roles of other inflammasomes besides NLRP3 in HD. In addition to abnormal protein aggregation, mitochondrial damage, lysosomal rupture, and autophagy dysfunction may also be inducers of inflammasome activation [123], all of which require further research.

### Inflammasomes in multiple sclerosis (MS)

MS is a chronic autoimmune disease of the nervous system. It is characterized by infiltration of peripheral immune cells, particularly T and B cells, into the central nervous system through compromised blood–brain barrier, where they attack and destroy the myelin sheath, accompanied by microglial activation, resulting in demyelinating lesions and neuroinflammation [124, 125]. Myelin regeneration refers to the process of restoring the insulating properties and function of nerve fibers by forming new myelin after demyelination. This process is particularly important for restoring myelin function. However, myelin regeneration is hindered in MS due to chronic neurological responses [126]. Experimental autoimmune encephalomyelitis (EAE) is the most widely used experimental model of MS, in which microglia and astrocytes play have dual roles. They may exert a neuroprotective effect in the early stages by removing damage and promoting repair in response to CNS injury. However, their long-term activation can lead to neurodegenerative changes [127]. Mature oligodendrocytes are responsible for the formation of myelin, to restore impaired nerve conduction caused by myelin destruction [128].

Inflammatory factors such as IL-17, IFN-γ, TNF-α and IL-6, and chemokines MIP-1a and CXCL9 in CSF and plasma, can serve as potential markers for MS, indicating the important role of neuroinflammation in MS [129–131]. Inflammasome-related markers such as NLRP3 protein, ASC, and caspase-1, as well as downstream IL-1β and IL-18, may also serve as potential biomarkers for MS [132]. The mRNA expression of *NLRP3* in PBMCs of MS patients increases over time [133]. Similarly, serum levels of caspase-1, ASC and IL-18 are elevated in MS patients, while IL-1β level is lower [134]. However, in primary progressive MS patients, IL-1β is highly expressed in monocytes from peripheral blood and CSF [135]. This suggests that the expression of IL-1β may not be completely the same in different subtypes of MS patients and may be regulated by different factors. Once released, IL-1β and IL-18 further promote T-cell responses and endothelial cell damage, exacerbating the severity of EAE [136]. In the EAE mouse model, increased levels of NLRP3 protein have been found not only in glial cells, but also in macrophages, T cells, and B cells [137]. In the hippocampus of EAE mice, both microglia and the NLRP3 inflammasome are activated, and treatment with an NLRP3 inhibitor can suppress the activation of both [138]. Studies have shown that Nlrp3^−/−^ mice are resistant to EAE induced by low-dose *Mycobacterium tuberculosis*, but susceptible to EAE under high-dose *Mycobacterium tuberculosis*. This suggests that different doses may induce EAE through different mechanisms and NLRP3 may play different roles in different immune environments [139]. NLRP3-knockout EAE mice show reductions of neutrophil infiltration, ROS production and recruitment of Th1 and Th17 cells compared to WT EAE mice [140]. In addition, inhibition of Th17 cells may reduce NLRP3 inflammasome activation [141]. Another study has shown that inhibiting the level of IL-1β in MS patients can have a symptom-relieving effect, but this is based on MS patients who are dependent on the NLRP3 inflammasome [142], which also suggests that only when the NLRP3 inflammasome is activated in MS patients or EAE models can inhibition of IL-1β have the effect of relieving MS symptoms. Research has shown that treatment of EAE animals with a caspase-1 inhibitor improves neurobehavioral performance, reduces the severity of neuropathology, and decreases inflammatory molecular markers. This suggests that caspase-1 is involved in the pathology of MS [143]. In addition, experiments have shown that targeting ASC can effectively inhibit the cleavage of caspase-1 and secretion of IL-1β, thereby alleviating EAE symptoms [144]. This result suggests that inhibition of ASC can alleviate EAE and provides evidence for the involvement of ASC in MS. Research has shown that in patients with relapsing–remitting MS (RRMS), IL-11 is most highly expressed in myeloid cells, particularly dendritic cells, neutrophils and monocytes, participating in the disruption of the blood–brain barrier and promoting migration of inflammatory cells into the CNS. Meanwhile, caspase-1 and IL-18 gene expression induced by the NLRP3 inflammasome in PBMCs is also increased in RRMS patients [145]. The levels of IL-1β, IL-18, caspase-1, and NLRP3 can be effectively reduced through Nrf2 activation and NF-kB pathway inhibition, thereby reducing ROS generation and damage [146, 147]. Meanwhile, inhibition of the p38 MAPK pathway can also prevent the neuroinflammation and demyelination caused by EAE [148]. This evidence suggests that the NLRP3 inflammasome may play a certain pathological role in MS, but is not activated in all subtypes of MS. More detailed research is needed to unravel the differences among MS subtypes.

In addition to NLRP3 activation in MS, the roles of other inflammasomes in MS are not yet clear. In the PBMCs of MS patients, the gene levels of NLRP1, NLRC4, and AIM2 were not elevated [149]. However, another study found that the levels of NLRC4 were increased in EAE and the brains of MS patients after death [143]. In addition, in MS patients, treatment with interferon β 1a significantly decreased mRNA expression of NLRP3, NLRC4 and AIM2 in leukocytes as well as plasma level of IL-1β [150]. In late EAE, the AIM2 inflammasome is activated in astrocytes, which may be independent of IL-1β-mediated inflammation [151]. Genetic research has shown that specific variations in *NLRP3* may lead to excessive production of IL-1β and IL-18, while reduced activation of NLRC4 or decreased production of IL-18 may be beneficial in MS patients [152]. One study found that NLRP12^−/−^ EAE mice had earlier and more severe clinical and pathological EAE outcomes, suggesting that the absence of NLRP12 leads to increased inflammatory responses in microglia [153]. After EAE induction, mRNA expression of *GSDMD* is increased [154]. A more recent study showed increased expression of GSDMD in both oligodendrocytes and microglia in the brain tissues of patients with progressive multiple sclerosis (P-MS), and *GSDMD* knockout in a cuprizone (CPZ)-induced demyelination mouse model reduced demyelination and neuronal axon damage [155].

Ion channels also play a critical role in MS. For example, the transient receptor potential vanilloid 1 (TRPV1) channel modulates ATP-induced NLRP3 inflammasome activation by mediating Ca^2+^ influx and phosphorylation of the phosphatase PP2A (protein phosphatase 2A) [156]. *TRPM2* (transient receptor potential melastatin 2) deletion can protect mice from CPZ-induced demyelination, synaptic loss, microglial activation, NLRP3 inflammasome activation, and pro-inflammatory cytokine production, ultimately ameliorating cognitive decline [157]. Inhibition of the TRPV4 channel ameliorated demyelination and inhibited glial activation and production of TNF-α and IL-1β in a CPZ-induced demyelination mouse model [158]. Another study suggests that although inhibition of the TRPV4 channel may reduce microglial activity and have anti-inflammatory effects, it does not significantly alter the severity of MS [159]. Some evidence suggests that high-mobility group protein 1 (HMGB1) contributes to MS through TLRs, and inhibition of HMGB1 can reduce inflammatory responses and improve cognitive and motor functions in MS patients [160]. These studies suggest that inhibiting downstream pathways of inflammation may also be a strategy for alleviating MS.

### Inflammasomes in ALS

ALS is a fatal neurodegenerative disease of the central nervous system. Most ALS patients have a hereditary component, with mutations in *C9orf72*, *TARDBP* (also known as TDP43), *SOD1 *(superoxide dismutase 1), and *FUS2* (Fused in sarcoma 2) accounting for the vast majority of cases [161]. Of these, the TDP43 and SOD1 proteins are the most extensively studied, and transgenic animals based on TDP43 and SOD1 are widely used to study ALS [162]. TDP43 and mutant SOD1 form abnormal aggregates in the cytoplasm of neurons, which impair mitochondrial function, autophagy, and the ubiquitin–proteasome system, leading to impaired protein clearance and subsequently affecting neuronal function [162, 163]. Increased levels of NLRP3, ASC, IL-18, and activated caspase-1 have been detected in the spinal cord tissues of ALS patients compared to controls [164]. Similarly, in the brains of ALS patients, the level of NLRP3 protein is increased compared to the control group [165]. In the serum and CSF of ALS patients, IL-6 and IL-18 are increased while IL-37 is absent, suggesting that IL-6 and IL-18 are involved in the pathological progression of ALS and can be used as inflammatory markers for ALS [166].

Studies have shown that in ALS, SOD1 and TDP43 can directly activate the NLRP3 inflammasome in microglia, leading to caspase-1-dependent inflammasome activation and production of IL-1β [167]. TDP43 interacts with the CD14 receptor on the surface of microglia, triggering the NF-κB pathway and NLRP3 inflammasome activation, leading to production of NOX2 (nicotinamide adenine dinucleotide phosphate oxidase 2), TNF-α, and IL-1β [168]. In the SOD1^G93A^ ALS mouse model, significantly increased immunoreactivity for ASC and NLRP3 was observed, suggesting the NLRP3 inflammasome activation in ALS [169]. Similarly, in SOD1^G93A^ mice, the level of *NLRP3* expression positively correlates with lifespan, suggesting that NLRP3 may contribute to muscle repair in the early stages of the disease. In the later stages of the disease, significant increases of protein levels of inflammasome components are observed, and mature IL-1β and active caspase-1 are detected in SOD1^G93A^ mice, suggesting NLRP3 inflammasome activation in skeletal muscles of the ALS mouse model, potentially promoting the pathological process [170]. Mutant SOD1 can lead to increased oxidative stress, and the increased ROS may promote aggregation and activation of inflammasome components [171]. Studies have shown that inhibiting activation of the NF-κB pathway in microglia can decrease levels of NO and ROS, inhibit protein nitration and inflammasome activation, and improve motor deficits in SOD1^G93A^ mice [172, 173]. In the spinal cord of SOD1^G93A^ mice, inhibition of IL-6 and caspase-1 can effectively reduce neuroinflammatory responses [174]. Similarly, inhibition of caspase-1 can attenuate glial cell activation and reduce neuroinflammation in SOD1^G93A^ mice [175]. Research has shown that P2X7 receptor antagonists can improve motor function and muscle strength in SOD1^G93A^ mice by inhibiting inflammasome assembly and reducing IL-1β release [176].

In the SOD1^G93A^ transgenic mice and in pathological samples from human ALS patients, the expression level of receptor-interacting serine/threonine-protein kinase 1 (RIPK1) is increased, which is associated with inflammation and axonal degeneration. This suggests that RIPK1 plays an important role in the inflammatory process of ALS [177]. Abnormal accumulation of TDP43 protein and mitochondrial dysfunction may lead to the release of mtDNA, thereby activating the cGAS-STING pathway and triggering an inflammatory response. The inflammatory factors produced after activation of the cGAS/STING pathway can enhance the inflammasome activation [178]. MCP-1 produced under the stimulation of TDP43 binds to the CC chemokine receptor 2, which further promotes migration and aggregation of monocytes and macrophages to the site of inflammation; these cells can further contribute to inflammasome activation [179]. In addition to microglial involvement, astrocytes release the pro-inflammatory factors TNF-α and IL-1β, causing damage to motor neurons in ALS patients [180]. Glycoprotein non-metastatic melanoma protein B (GPNMB), a transmembrane glycoprotein that has been shown to prolong the survival of SOD1^G93A^ mice, has been found to exert its neuroprotective effect through reducing astrocyte-mediated neuroinflammation in a CD44-dependent manner [181].

In a recent study, acute treatment with an NLRP3-specific inhibitor did not reduce spinal cord inflammation in SOD1^G93A^ mice, suggesting that multiple inflammasomes may be activated in ALS and that inhibition of NLRP3 alone may not be sufficient to block inflammation [182]. The expression of NLRP1 is downregulated in SOD1^G93A^ mice, but no such changes have been observed in ALS patients, while the protein levels of NLRC4 and AIM2 are increased in symptomatic animals [183]. However, another study found that the mRNA expression of NLRP1b and NLRC4 was significantly increased in symptomatic SOD1^G93A^ mice, while AIM2 did not change significantly. In ALS patients, the protein level of NLRC4 is significantly increased, while AIM2 is decreased but without statistical significance [184]. In ALS patients with cognitive impairment, only *NLRP3* expression is upregulated in neurons and glial cells compared to those without cognitive dysfunction [185]. GSDMD expression has been found in the TDP43 mouse model and in the motor cortex and spinal cord of ALS patients, suggesting that the inflammasome triggers pyroptosis activation in ALS [186]. These studies indicate the need for further investigations of the specific pathological mechanisms of inflammasomes in ALS.

## Inflammasomes as pharmacological targets

Inflammasomes play a critical role in neurodegenerative diseases, leading to the discovery or development of many compounds targeting them for pharmacological intervention (Table 1). The NLRP3 inflammasome is a main pharmacological target. Currently, the most widely used and gold-standard compound for NLRP3 inhibition is MCC950 (also known as CRID3). MCC950 is a specific inhibitor that can block both canonical and non-canonical pathways of NLRP3 inflammasome activation. It has been shown to specifically inhibiting NLRP3 without suppressing AIM2, NLRC4 or NLRP1 inflammasome activation. In addition, MCC950 can effectively cross the blood–brain barrier and has been experimentally tested for neurodegenerative diseases [187]. Glyburide is a sulfonylurea drug commonly used to treat type-2 diabetes. It works by inhibiting ATP-sensitive potassium channels in pancreatic β-cells. Glyburide also inhibits NLRP3, caspase-1, and IL-1β in macrophages and may be used as an inflammasome inhibitor to treat NLRP3-related inflammatory diseases [188]. CY-09 is an inhibitor that specifically blocks the NLRP3 inflammasome activation. It works by binding directly to the ATP-binding site of the NACHT domain of NLRP3, inhibiting its ATPase activity, thereby preventing NLRP3 oligomerization and inflammasome assembly [189]. CY-09 and its related analogues have been used to inhibit the activation of NLRP3 in monocytes and subsequently applied in AD. More studies are needed to determine whether they can be applied to other neurodegenerative diseases [190]. OLT1177, TRANILAST, ORIDONIN, JC171 and MNS all inhibit NLRP3 inflammasome activation yet via different mechanisms, and are applicable to only a few neurodegenerative diseases. Other inflammasome inhibitors, such as BAY 11–7082 and PARTHENOLIDE, can target NLRP3 by inhibiting its atpase activity. However, they are not specific for NLRP3, as they can also target AIM2, NLRC4, and NLRP1 [191], suggesting diverse effects of these inhibitors. However, in AD, BAY 11–7082 exerts its effects by inhibiting NLRP3, but not AIM2, NLRC4, or NLRP1 [192]. Other NLRP3 inhibitors such as BOT-4-one and INF39 have not been tested in neurodegenerative disease models.

**Table 1:** Overview of pharmacological compounds targeting inflammasome

Compound name	Mechanism of action	Specificity	Applicable diseases	Refs
MCC950	Interacts with the Walker B motif within the NACHT domain of NLRP3, preventing ATP hydrolysis	NLRP3	AD, PD, MS, HD	[91, 120, 213, 214]
Glyburide	ATP sensible K^+^ channels, downstream of the P2X7 receptor	NLRP3	AD, PD	[188, 215]
CY-09	Binds to the ATP-binding region of NLRP3 protein and inhibits the ATP hydrolytic activity of NLRP3	NLRP3	AD	[190]
OLT1177	Binds to NLRP3 and inhibits its ATPase activity	NLRP3	AD, PD, MS	[216–218]
TRANILAST	Interacts with the NLRP3 NACHT domain	NLRP3	AD	[219]
ORIDONIN	Inhibits recruitment of NEK7 by covalently modifying Cys279 in the NLRP3 NACHT domain	NLRP3	AD	[220]
JC171	Inhibits the NLRP3 activation	NLRP3	AD, PD, MS	[221, 222]
MNS	Blocks NLRP3-mediated ASC speck formation and oligomerization	NLRP3	AD, PD	[223]
BAY 11–7082	Inhibits NACHT ATPase activity	NLRP3, NLRC4	AD, PD, MS	[192, 224, 225]
PARTHENOLIDE	Inhibits NACHT ATPase activity	NLRP3, NLRC4, NLRP1, AIM2	AD, PD, MS	[189, 226–228]
VX765	Inhibits caspase-1	Caspase-1	AD, PD, MS	[143, 193, 229]
Indomethacin	Inhibits the expression of IL-1β and caspase-1	IL-1β, Caspase-1	AD, PD	[230, 231]
Anakinra	IL-1 receptor antagonist	IL-1	AD, PD, MS, ALS	[196, 208, 232, 233]
BHB	Inhibits NLRP3 inflammasome activation and reduces ASC oligomerization and speck formation	NLRP3, ASC	AD, PD, MS, HD, ALS	[234–238]
IC100	Inhibits ASC oligomerization	ASC	AD, MS	[194, 239]
Disulfiram	Modifies and inhibits GSDMD	GSDMD	PD, MS	[199, 240]
Necrosulfonamide	Modifies and inhibits GSDMD	GSDMD	AD, PD	[200, 241]
Dimethyl fumarate	Modifies and inhibits GSDMD	GSDMD	AD, PD, MS, HD, ALS	[202, 206, 242–244]

In addition to directly inhibiting NLRP3 initiation and assembly, there are inhibitors that target other components of the inflammasome. For example, VX740 and VX765 selectively inhibit caspase-1, which can block the secretion of IL-1β and IL-18, thereby reducing inflammatory responses. Inhibition of caspase-1 has been shown to effectively ameliorate cognitive impairment in AD mice [193], suggesting that caspase-1 inhibitors have potentials to be used for neurodegenerative diseases. ASC is an adaptor protein that not only facilitates the assembly of the inflammasome, but also affects extracellular inflammation, and macrophages can uptake and release ASC plaques, leading to the persistence of inflammatory response. Research has shown that targeting ASC within the inflammasome can also inhibit inflammatory responses [21]. IC100, a monoclonal antibody that inhibits ASC oligomerization, has been found to attenuate inflammatory responses in the EAE model and exert considerable therapeutic potential, suggesting potentials of ASC inhibitors for neurodegenerative diseases [194]. Some inhibitors target downstream effectors of the inflammasome, such as IL-1, to suppress inflammation. Canakinumab (Ilaris), rilonacept (Arcalyst), and anakinra (Kineret) are three IL-1 antagonists that have been approved for clinical use [195]. Of these, anakinra has been used in various neurodegenerative disease models in vitro and in vivo, and has demonstrated safety when used to block IL-1 in ALS patients [196].

Pyroptosis is a form of programmed cell death that triggers a robust inflammatory response. GSDMD is a key mediator of pyroptosis. GSDMD can be cleaved by inflammatory caspases, releasing its active amino-terminal fragment. This fragment translocates to the plasma membrane, undergoes conformational changes, and oligomerizes to form a pore-like structure on the membrane. These pores allow the cell to release a large amount of pro-inflammatory cytokines, causing a strong inflammatory response and inducing pyroptosis of immune cells [197, 198]. Therefore, inhibiting GSDMD to reduce inflammatory responses may also be a strategy. Disulfiram is a blocker of GSDMD pore formation and has been shown to reduce inflammation in a cell model of PD. Changes in GSDMD have also been found in the blood of PD patients, suggesting that inhibition of GSDMD may alleviate neurodegenerative diseases [199]. Necrosulfonamide, an inhibitor with a similar mechanism as disulfiram, has been found to reduce neuroinflammation and rescue dopaminergic neuronal degeneration in the MPTP model [200]. Dimethyl fumarate has been approved for the treatment of MS in the clinic to reduce inflammatory responses in MS [201]. However, another study found that dimethyl fumarate treatment in the EAE model alleviated inflammatory response after one week of treatment, but it did not ameliorate neuroinflammation and microglial activation after two weeks of treatment [202]. These results suggest that GSDMD-dependent inflammasome-mediated pyroptosis may be a potential target for treating neurodegenerative diseases. Ninjurin-1 (NINJ1) is a cell membrane surface protein also associated with pyroptosis. Some studies have shown that inhibiting NINJ1 can reduce cell damage [203]. However, there is little evidence to date for its application in neurodegenerative diseases.

There are some inhibitors that have already undergone clinical trials for ulcerative colitis, acute gout attacks, osteoarthritis, cytokine release syndrom in COVID-19, cryopyrin-associated periodic syndrome, and advanced cancer (combined therapy) (Table 2). Most of the trials tested NLRP3 inflammasome inhibitors with a primary aim of reducing inflammatory factors. There are relatively few clinical trials for neurodegenerative diseases. ZYIL1 can inhibit the NLRP3 pathway by blocking ASC oligomerization [204]. In the clinical trial NCT05981040, ZYIL1 was applied to ALS subjects to assess its safety, tolerability, pharmacokinetics, and pharmacodynamics during administration. The pharmacodynamic data can be used to determine whether the NLRP3 inhibitor ZYIL1 can improve ALS symptoms. Colchicine is an anti-inflammatory drug primarily used for the treatment of gout. In recent years, it has been found to inhibit inflammasome activation [205]. In the clinical trial NCT03693781, Colchicine was used to enhance autophagy to reduce TDP-43 accumulation in neurons, and alleviate ALS symptoms through its anti-inflammatory function. Dimethyl fumarate can inhibit the interaction between NLRP3 and NEK7, and by modifying GSDMD, it plays a role in inhibiting the NLRP3 inflammatory pathway [206]. The clinical trials NCT02675413 and NCT02461069 assessed the efficacy of dimethyl fumarate in alleviating MS symptoms, and its possible mechanism of action is to enhance Nrf2 transcription and exert anti-inflammatory effects [207]. Similarly, antibodies for IL-1β, which have been approved for the treatment of neurodegenerative diseases, have demonstrated significant individual differences in efficacy, and long-term safety has not been fully verified [208], weakening their potential for clinical use. These drugs mentioned above are used for palliative treatment of patients to alleviate symptoms, and the scope of treatment is narrower. At the same time, for diseases like AD and PD, where there is substantial evidence that inflammasomes play an important role, there are fewer related clinical trials.

**Table 2:** Inflammasome inhibitors in clinical development

Compound name	Development stage	Conditions
ZYIL1	Phase 2 (NCT05981040)	Amyotrophic lateral sclerosis
	Phase 2 (NCT06398808)	Ulcerative colitis
	Phase 2 (NCT05186051)	Cryopyrin associated periodic syndrome
OLT1177	Early Phase 1 (NCT05880355)	Myocardial infarction
	Phase 2 (NCT05658575)	Acute gout flare
	Phase 2 (NCT04540120)	COVID-19, cytokine release syndrome
	Phase 2 (NCT01768975)	Osteoarthritis of the knee
	Phase 2 (NCT06047262)	Diabetes mellitus, type 2
DFV890	Phase 1 (NCT05552469)	Myeloid diseases
	Phase 2 (NCT04868968)	Familial cold autoinflammatory syndrome
	Phase 2 (NCT04886258)	Symptomatic knee osteoarthritis
	Phase 2 (NCT06031844)	Coronary heart disease
RRx-001	Phase 2 (NCT03515538)	Oral mucositis
	Phase 2 (NCT05966194)	Oral mucositis
Inzomelid	Phase 1 (NCT04015076)	Cryopyrin associated periodic syndrome
VTX2735	Phase 2 (NCT05812781)	Cryopyrin associated periodic syndrome
NT-0796	Phase 2 (NCT06129409)	Cardiovascular diseases
Tranilast	Phase 2 (NCT00882024)	Active rheumatoid arthritis
	Phase 2 (NCT03923140)	Cryopyrin associated periodic syndrome
BMS-986299	Phase 2 (NCT03444753)	Advanced cancer
Oridonin	Phase 4 (NCT05130892)	Percutaneous coronary intervention
Colchicine	Phase 3 (NCT06054100)	Acute coronary syndrome
	Phase 2 (NCT03693781)	Amyotrophic lateral sclerosis
	Phase 3 (NCT05855746)	Acute myocarditis
Dimethyl fumarate	Phase 4 (NCT02675413)	Multiple sclerosis
	Phase 4 (NCT02461069)	Multiple sclerosis, relapsing–remitting

Data searched from clinicaltrials.gov.

## Conclusion

Inflammasomes play a critical role in the innate immune system by recognizing PAMPs or host-derived DAMPs and are essential for host protection. However, excessive or prolonged inflammasome activation can trigger inflammatory responses. Inflammasome activation depends on PAMPs and DAMPs, such as misfolded proteins, increased kinases, gut microbiota dysbiosis, or the release of ATP. It is also possible to directly activate or inhibit inflammasomes by modulating these PAMPs and DAMPs. Additionally, inhibiting key molecular mechanisms upstream and downstream of inflammasome activation can achieve the same purpose. Beyond this, regulating autophagy can also regulate inflammasome activation. Existing research has found that autophagy can inhibit NLRP3 inflammasome activation in three ways, including degradation of ASC, increasing NLRP3 phosphorylation, and clearing ROS [209]. Similarly, oxidative stress, mitochondrial-related factors, and ion channels can also be used as targets to regulate inflammasome acivation[210–212].

In neurodegenerative diseases, inflammasome activation, particularly the NLRP3 inflammasome activation, has been extensively studied for its role in AD and PD, but its specific pathogenic mechanisms in HD and ALS are not well understood. In addition to NLRP3, other inflammasomes such as AIM2, NLRC4 and NLRP1 and their downstream effector molecules have been found to play pathological roles in neurodegenerative diseases. However, more preclinical experiments and clinical trials are needed to confirm these findings. Understanding the complex interactions between inflammasomes and the nervous system is essential for identifying potential therapeutic targets and developing effective treatments for neurodegenerative diseases.

Given the role of inflammasomes in neurodegenerative diseases, targeting these complexes has emerged as a potential therapeutic strategy. Some small molecules that inhibit inflammasome activation or assembly have been experimentally used to alleviate neuroinflammation in these diseases. However, there are also small molecules that cannot cross the blood–brain barrier and are therefore not applicable to these diseases. This highlights the need to focus on compounds with both inhibitory potential and potential for modification, with the goal of identifying compounds that can modulate inflammasome activation without causing side effects or off-target activities. Targeting downstream effector molecules of inflammasomes, such as IL-1β and IL-18, or targeting GSDMD, which is associated with inflammasome-induced pyroptosis, is a potential strategy. In addition, some drugs with other clinical uses, such as nonsteroidal anti-inflammatory drugs, have been found to inhibit inflammasome activation, suggesting their potential of re-direction. Another strategy is to develop inhibitors that target multiple pathways, such as combinational therapies that simultaneously target different aspects of the inflammatory process, potentially providing more effective control of neuroinflammation in neurodegenerative diseases. In summary, targeting inflammasomes and their associated pathways holds great promise for the treatment of neurodegenerative diseases. However, this approach requires careful considerations of compound specificity, ability to cross the blood–brain barrier, and the overall impact on disease progression.

## Data Availability

Not applicable.

## Abbreviations

AAV: Adeno-associated virus
AD: Alzheimer’s disease
AIM2: Absent in melanoma 2
ALR: AIM2-like receptors
ALS: Amyotrophic lateral sclerosis
AMPK: Adenosine 5’-monophosphate-activated protein kinase
ASC: Apoptosis-associated speck-like protein
Aβ: Amyloid-β
Calhm2: Calcium homeostasis modulator 2
CNS: Central nervous system
CPZ: Chlorpromazine
CSF: Cerebrospinal fluid
CXCL9: Chemokine CXC ligand 9
DAM: Disease-associated microglia
DAMPS: Damage associated molecular patterns
EAE: Experimental autoimmune encephalomyelitis
FUS2: Fused in sarcoma 2
Fyn: FYN proto-oncogene, Src family tyrosine kinase Gene
GSDMD: Gasdermin D
HD: Huntington’s disease
HMGB1: High-mobility group box 1
IFN-γ: Interferon-γ
IL: Interleukin
INPP5D: Inositol Polyphosphate-5-phosphatase D
MPTP: 1-Methyl-4-phenyl-1,2,3,6-te-trahydropyridine
MS: Multiple Sclerosis
mTOR: Mammalian target of rapamycin
NFTs: Neurofibrillary tangles
NF-κB: Nuclear factor-kappa B
NINJ1: Nerve injury-induced protein 1
NLRC4: Nodlike receptor and caspase recruitment domain-containing protein 4
NLRP1: Nod-like receptor and pyrin domain-containing protein 1
NLRs: Nucleotide oligomerization domain (NOD)-like receptors
Nrf2: Nuclearrespiratoty factor 2
PAMP: Pathogen-associated molecular pattern
PBMC: Peripheral blood mononuclear cell
PD: Parkinson’s disease
PFF: Pre-formed fibrils
PIPs: Phosphatidylinositol phosphates
PRRs: Pattern recognition receptor
RIPK1: Receptor-interacting serine/threonine-protein kinase 1
ROS: Reactive oxygen species
SGK1: Serum and glucocorticoid-induced protein kinase 1
SHIP1: Src homology 2-containing inositol phosphatase-1
SNpc: Substantia nigra pars compacta
SOD1: Superoxide dismutase 1
TDP43: TAR DNA binding protein-43
TLRs: Toll-like receptors
TNF: Tumor necrosis factor
TREM2: Triggering receptor expressed on myeloid cells 2
TRPV1: Transient receptor potential vanilloid 1
α-Syn: α‐Synuclein
