# Correction: Inflammasomes in neurodegenerative diseases

**DOI:** 10.1186/s40035-025-00468-7

**Published:** 2025-01-23

**Authors:** Qianchen Wang, Songwei Yang, Xuan Zhang, Shanshan Zhang, Liping Chen, Wanxue Wang, Naihong Chen, Jiaqing Yan

**Affiliations:** 1 Department of Pharmacy, The First Affiliated Hospital of China Medical University, Shenyang, 110001 China; 2 Hunan Engineering Technology Center of Standardization and Function of Chinese Herbal Decoction Pieces, School of Pharmacy, Hunan University of Chinese Medicine, Changsha, 410208 China; 3 Department of Pharmacy, National Cancer Center/National Clinical Research Center for Cancer/Cancer Hospital, Chinese Academy of Medical Sciences and Peking Union Medical College, Beijing, 100021 China; 4 China Three Gorges University College of Medicine and Health Sciences, Yichang, 443002 China; 5 State Key Laboratory of Bioactive Substances and Functions of Natural Medicines, Institute of Materia Medica and Neuroscience Center, Chinese Academy of Medical Sciences and Peking Union Medical College, Beijing, 100050 China

**Correction: Translational Neurodegeneration (2024) 13:65** 10.1186/s40035-024-00459-0

Following publication of the original article [1], the authors reported an error in corresponding author’s affiliation. The full list of authors’ affiliations is corrected from:

Qianchen Wang^1†^, Songwei Yang^2†^, Xuan Zhang^3^, Shanshan Zhang^4^, Liping Chen^1^, Wanxue Wang^5^, Naihong Chen^5^ and Jiaqing Yan^1,3*^

1 Department of Pharmacy, The First Affiliated Hospital of China Medical University, Shenyang 110001, China

2 Hunan Engineering Technology Center of Standardization and Function of Chinese Herbal Decoction Pieces, School of Pharmacy, Hunan University of Chinese Medicine, Changsha 410208, China

3 Department of Pharmacy, National Cancer Center/National Clinical Research Center for Cancer/Cancer Hospital, Chinese Academy of Medical Sciences and Peking Union Medical College, Beijing 100021, China 

4 China Three Gorges University College of Medicine and Health Sciences, Yichang 443002, China

5 State Key Laboratory of Bioactive Substances and Functions of Natural Medicines, Institute of Materia Medica and Neuroscience Center, Chinese Academy of Medical Sciences and Peking Union Medical College, Beijing 100050, China

To:

Qianchen Wang^1†^, Songwei Yang^2†^, Xuan Zhang^3^, Shanshan Zhang^4^, Liping Chen^1^, Wanxue Wang^5^, Naihong Chen^5^ and Jiaqing Yan^3*^

1 Department of Pharmacy, The First Affiliated Hospital of China Medical University, Shenyang 110001, China

2 Hunan Engineering Technology Center of Standardization and Function of Chinese Herbal Decoction Pieces, School of Pharmacy, Hunan University of Chinese Medicine, Changsha 410208, China

3 Department of Pharmacy, National Cancer Center/National Clinical Research Center for Cancer/Cancer Hospital, Chinese Academy of Medical Sciences and Peking Union Medical College, Beijing 100021, China 

4 China Three Gorges University College of Medicine and Health Sciences, Yichang 443002, China

5 State Key Laboratory of Bioactive Substances and Functions of Natural Medicines, Institute of Materia Medica and Neuroscience Center, Chinese Academy of Medical Sciences and Peking Union Medical College, Beijing 100050, China

The original article [1] has been updated.
